# Development and validation of high-dose methotrexate population pharmacokinetic models to inform clinical decisions on dosing

**DOI:** 10.1007/s00228-026-04080-0

**Published:** 2026-05-23

**Authors:** Marisa H. Blackman, Bradley Yelvington, Cole Beck, Manuel Cortez, Leena Choi

**Affiliations:** 1 Department of Biostatistics, Vanderbilt University Medical Center, Nashville, TN USA; 2 Department of Oncology Pharmacy, Vanderbilt University Medical Center, Nashville, TN USA; 3 Department of Pharmacy, Rush MD Anderson Cancer Center, Chicago, IL USA

**Keywords:** Pharmacokinetics, Pharmacometrics, Model evaluation, Cancers

## Abstract

**Purpose:**

High-dose methotrexate is an effective treatment for adult and pediatric patients with acute lymphoblastic leukemia, osteosarcoma, and lymphoma. However, its clearance is highly variable, and delayed clearance can lead to significant toxicity. This study aimed to identify a population pharmacokinetic model that accurately characterizes high-dose methotrexate clearance in an adult population.

**Methods:**

We developed a new population pharmacokinetic model using a training dataset derived from a cohort of 208 adult patients who received high-dose methotrexate at Vanderbilt University Medical Center. To assess predictive performance, we evaluated both our model and several externally developed models using an independent test dataset.

**Results:**

The final model was a three-compartment model incorporating body surface area as a covariate on all pharmacokinetic parameters, and serum creatinine and sex as covariates on clearance. Our newly developed model predicted with the most accuracy and precision at the first concentration measurement, taken at 24 h. Our model, along with five external models, was selected based on predictive performance for further assessment with maximum a posteriori Bayesian forecasting to compare predictions at the individual-level. The model by Hui et al. was more accurate at 48 h, while our model performed similarly at 72 h.

**Conclusion:**

These findings suggest an optimal strategy for therapeutic drug monitoring and dosing decisions: use our model for prediction at the 24 h mark when no prior drug levels are available, followed by Bayesian forecasting using our new model supplemented by the Taylor model.

**Supplementary Information:**

The online version contains supplementary material available at https://doi.org/10.1007/s00228-026-04080-0.

## Introduction

Methotrexate (MTX) is a folate antimetabolite commonly used to treat autoimmune diseases and various cancers [1]. High-dose MTX (HD-MTX) is effective in treating adult and pediatric patients with acute lymphoblastic leukemia (ALL), osteosarcoma (OS), and lymphoma. However, it has significant risks. Delayed clearance of HD-MTX is associated with toxicity of the liver, kidneys, and gastrointestinal tract. Moreover, managing toxicities from HD-MTX is challenging due to its highly variable inter-individual clearance [2]. Glucarpidase, a rescue drug, is available for emergency use when MTX concentrations exceed recommended levels. However, the clinical guidelines for glucarpidase administration are often unclear to clinicians [3]. Additionally, the high cost of glucarpidase makes avoiding it preferable when possible. To address these challenges, it is critical to predict clearance in real-time during treatment, which can be supported with therapeutic drug monitoring (TDM) tools.

Recently, a freely available online TDM tool based on a HD-MTX population pharmacokinetic (popPK) model was developed to help clinicians make informed post-infusion monitoring decisions [3]. The model (referred to here as the Taylor model) was originally developed using data from pediatric patients in Nordic countries. Although later validated in an adult cohort [4], it may not perform well in our adult cohort due to potential differences in patient characteristics. Accurate personalized dosing via TDM requires a reliable underlying popPK model.

In addition to the Taylor model, several popPK models for HD-MTX have been published. A systematic review of 30 models evaluated their predictive performance [5]. Due to high inter-individual variability in clearance, externally developed models may struggle to achieve adequate predictive performance across patient populations due to differences in factors influencing clearance, including body size (weight, body surface area [BSA]) [3, 6], renal function (serum creatinine [SCR], blood urea nitrogen, estimated glomerular filtration rate [eGFR]) [7, 8], age [8–10], and genetic/regional factors [2, 11].

Model validation typically involves identifying a model, generating predictions, and calculating predictive performance metrics. Commonly used metrics include R-squared (R^2^), root mean squared error (RMSE), percent prediction error (PE%), median percent prediction error (MPE), and median absolute percent prediction error (MAPE). More recently, F20 and F30 have been introduced for external validation, representing the proportion of PE% values within ± 20% and ± 30%, respectively [12]. These methods are most effective for comparing multiple external models, as there is no objective threshold for defining what constitutes a sufficiently good RMSE or PE%. This contrasts with traditional popPK model fitting procedure, where models can be compared using statistical criteria such as the objective function value (OFV) and Bayesian information criterion (BIC).

The goal of this study was to identify a popPK model that accurately characterizes HD-MTX clearance in an adult patient population. We evaluated several externally developed HD-MTX popPK models, including the Taylor model, to assess their predictive performance in a cohort of adult patients receiving HD-MTX at Vanderbilt University Medical Center (VUMC). We also developed a new popPK model using our patient data and compared its performance to the external popPK models using multiple evaluation metrics.

## Materials and methods

### Study design and data collection

This study was approved by the VUMC Institutional Review Board. Data were extracted from electronic health records (EHRs) for adult patients who received an HD-MTX containing regimen at VUMC between November 2017 and December 2022. Patients were identified by treatment protocols and included if they received at least one intravenous infusion (IV) MTX dose of 200 mg/m^2^ or higher. Dosing intervals were typically 2–4 weeks (median 3.4). Infusions were administered as short-term single doses (59%), long-term single doses (19%), or loading dose plus maintenance dose (22%). MTX concentrations were measured per institutional protocol, beginning 24 h after start of infusion and repeated every 24 h until the concentration dropped below 0.1 µmol/L. Leucovorin was initiated in all patients 24 h after MTX dosing as per institutional protocol. In the case of delayed clearance or concern for changing renal function, MTX levels were obtained earlier and/or more frequently and leucovorin dose and/or frequency were increased at the discretion of the provider. Patients who received glucarpidase were excluded, as it alters MTX clearance.

## Data and processing

EHR data were processed using the *EHR* R package within the *EHR2PKPD* system [13], which consolidates relevant information into a format suitable for popPK analysis [14]. SCR concentrations, typically measured concurrently with MTX concentrations, were used as a time-varying covariate. If an SCR measurement was not available at the time of MTX sampling, the closest available value was used. SCR values were originally provided in mg/dL and were converted to µmol/L. Weight, height, and BSA varied over time and were also included as time-varying covariates in the popPK modeling. In contrast, age, which changed minimally over the data collection period, and sex were treated as non-time-varying covariates.

MTX concentrations measured beyond 96 h post-dose were excluded as data were sparse, derived from only a small number of patients, and typically fell below the quantification limit. The study, therefore, focused on accurately predicting concentrations within approximately 60 h post-dose, as glucarpidase has been shown to be most effective when administered within 48–60 h after the start of infusion [15]. Furthermore, there is limited evidence supporting the effectiveness of glucarpidase beyond 60 h [15]. Data quality was also reviewed, with attention to potential infusion line contamination. Individual measurements or entire treatment cycles were excluded when line contamination was suspected. Of 2,448 originally measured MTX concentrations, 53 (2.2%) were excluded through data processing, resulting in a dataset with 2,395 concentrations. This final dataset was split randomly into training and test sets using a 70:30 ratio.

## Population PK analysis

We conducted popPK analysis on our training dataset using Monolix 2024R^®^ [16] (Lixoft, France), applying the stochastic approximation expectation-maximization (SAEM) algorithm for parameter estimation. To identify the base model, we compared one-, two-, and three-compartment models, allowing for correlation between random effects. For each structural model, we evaluated residual error structures, including proportional, additive, and combined error models. After selecting a base model, weight (kg) and BSA (m^2^) were evaluated as covariates on all PK parameters due to their known association with PK. Then, using this adjusted model, we proceeded with covariate selection, evaluating the following pre-selected covariates: height (m), sex, age (years), and SCR (µmol/L). Continuous covariates were standardized to their median except for weight, which was standardized to 70 kg. Weight was modeled using fixed allometric scaling exponents of 0.75 or 1. The effect of BSA on clearance was estimated, whereas its effect on all other PK parameters was fixed with an exponent of 1. This approach was selected due to the lack of a clear physiological rationale for alternative BSA scaling and to preserve model parsimony. Candidate models were compared based on OFV, Akaike information criterion (AIC), and BIC.

After selecting the final model, we evaluated it using goodness-of-fit plots, residual plots, and visual prediction check (VPC), all generated using R version 4.4.1 (The R Foundation for Statistical Computing, Vienna, Austria) [17].

## Model validation

The test dataset was used to validate our model along with external models identified from the review paper by Yang et al. [5], including the Taylor model, as well as more recently published models. Selection was based on the availability of the requisite covariates in our dataset. As our approach prioritizes routinely available bedside clinical variables, we included only models that had height, sex, age, SCR, BSA, or weight as covariates. Models including patients receiving glucarpidase were excluded. From each model, fixed parameter estimates and model structures were used to predict MTX concentrations in Monolix 2024R^®^. The following metrics were calculated to compare population predictions: RMSE, PE%, MPE, MAPE, F20, and F30. RMSE and PE% are defined as:

$$\:{RMSE}_{ij}=\sqrt{\frac{{\sum\:}_{i=1}^{n}{\sum\:}_{j=1}^{{m}_{i}}{\left(ob{s}_{ij}-pre{d}_{ij}\right)}^{2}}{{\sum\:}_{i=1}^{n}{\sum\:}_{j=1}^{{m}_{i}}1}},$$

$$\mathrm{PE\%}_{ij}=\frac{{pred}_{ij}-{obs}_{ij}}{{obs}_{ij}}\times\:100,i=\{\mathrm{1,2},\dots\:,n\},j=\{\mathrm{1,2},\dots\:,{m}_{i}\},$$

where *n* is the number of subjects, $\:{m}_{i}$ is the number of measurements per subject, and $\:ob{s}_{ij}$ and $\:pre{d}_{ij}$ are the *j*^th^ observed and predicted concentrations for the *i*^th^ individual, respectively. Predictive performance was evaluated using the following criteria: MPE ≤ 20%, MAPE ≤ 30%, F20 ≥ 35%, and F30 ≥ 50%.

Models demonstrating promising predictive performance were re-fit to the test dataset to update the popPK parameters, accounting for potential differences in estimates due to variation in patient characteristics. Selection for re-fitting was based on a composite evaluation of all performance metrics. The updated models were assessed using the same metrics.

Maximum *a posteriori* Bayesian forecasting was performed in NONMEM^®^ version 7.5.0 (ICON Development Solutions, MD, San Antonio, USA) [18] to generate individual-level predictions for each of the re-fit models. Predictions were made at three time points per cycle, including only patients with sufficient data at each step. PK parameters were updated using (1) the first concentration, (2) the second concentration, or (3) both. Approach (1) predicted the second concentration, while (2) and (3) predicted the third concentration. MTX concentrations were typically measured at 24, 48, and 72 h post-dose with some variability in sampling times, including occasional early draws. The observed median (interquartile range) sampling times were 24.3 (24.0–24.8), 48.0 (47.7–48.3), and 71.8 (60.7–72.4) hours. The Bayesian forecasting performances were assessed using individual PE% (IPE%), median individual PE% (MIPE), median absolute individual PE% (MAIPE), and the proportions of IPE% values within ± 20% (IF20) and ± 30% (IF30).

## Results

### Study population and sample characteristics

The final cohort contained 208 patients that were randomly split into training and testing datasets using a 70:30 ratio. The training dataset was used to develop a new popPK model, while the test dataset was used to validate all popPK models including the newly developed model.

Patient characteristics in the training and test datasets, and the overall cohort, are summarized in Table 1. The datasets were generally comparable, with median ages of 56.0 and 55.0 years, median BSA of 1.97 m^2^ in both, and median SCR of 68.5 and 66.3 mmol/L. A slight sex imbalance was observed (60.0% female in training dataset versus 46.0% in test). Excluding loading doses, the median administered dose was 3.5 (range 0.2–12.1) g/m^2^ for the training dataset and 3.0 (range 0.2–12.0) g/m^2^ for the test dataset. Median infusion duration was 4.7 h in both datasets (ranges of 2.0-27.9 and 2.0–28.0 h, respectively). The median number of drug levels and doses per patient were 9 and 4 in both datasets.

**Table 1:** Characteristics of study population and data summary

	Train (*N* = 145)	Test (*N* = 63)	Overall (*N* = 208)
Age (years)			
Mean (SD)	51.8 (17.9)	51.1 (17.3)	51.6 (17.7)
Median [Min, Max]	56.0 [18.0, 84.0]	55.0 [18.0, 87.0]	55.5 [18.0, 87.0]
**Sex**			
Female	87 (60.0%)	29 (46.0%)	116 (55.8%)
Male	58 (40.0%)	34 (54.0%)	92 (44.2%)
**Weight (kg)**			
Mean (SD)	84.5 (20.3)	84.6 (21.1)	84.5 (20.5)
Median [Min, Max]	82.4 [42.4, 141.0]	84.1 [50.1, 189.0]	83.1 [42.4, 189.0]
**Height (cm)**			
Mean (SD)	170.0 (10.6)	170.0 (10.4)	170.0 (10.5)
Median [Min, Max]	170.0 [148.0, 189.0]	170.0 [150.0, 191.0]	170.0 [148.0, 191.0]
**Body Surface Area (m** ^**2**^ **)**			
Mean (SD)	1.97 (0.28)	1.97 (0.27)	1.97 (0.28)
Median [Min, Max]	1.97 [1.32, 2.66]	1.97 [1.43, 3.01]	1.97 [1.32, 3.01]
**Serum Creatinine (µmol/L)**			
Mean (SD)	72.5 (16.4)	69.4 (13.8)	71.5 (15.7)
Median [Min, Max]	68.5 [44.2, 132]	66.3 [47.3, 117]	68.1 [44.2, 132]
**Number of Drug Levels per Person**			
Mean (SD)	11.6 (10.2)	11.4 (8.2)	11.5 (9.6)
Median [Min, Max]	9.0 [2.0, 52.0]	9.0 [2.0, 41.0]	9.0 [2.0, 52.0]
**Number of Dosing Events per Person**			
Mean (SD)	4.0 (2.9)	4.0 (2.5)	4.0 (2.8)
Median [Min, Max]	4.0 [1.0, 13.0]	4.0 [1.0, 12.0]	4.0 [1.0, 13.0]
**Number of Cycles per Person**			
Mean (SD)	3.3 (2.6)	3.3 (2.2)	3.4 (2.5)
Median [Min, Max]	3.0 [1.0, 12.0]	3.0 [1.0, 12.0]	3.0 [1.0, 12.0]
**Drug Level (mg/L)** ^*a^			
*Overall*			
Mean (SD)	2.31 (7.79)	2.78 (9.83)	2.45 (8.46)
Median [Min, Max]	0.12 [0.02, 108.00]	0.11 [0.02, 177.00]	0.12 [0.02, 177.00]
*1st concentration*			
Mean (SD)	7.54 (13.12)	9.05 (16.68)	8.00 (14.29)
Median [Min, Max]	4.21 [0.16, 108.04]	3.97 [0.05, 176.65]	4.11 [0.05, 176.65]
*2nd concentration*			
Mean (SD)	0.32 (0.66)	0.45 (1.50)	0.36 (0.99)
Median [Min, Max]	0.12 [0.02, 5.28]	0.14 [0.03, 13.80]	0.13 [0.02, 13.8]
*3rd concentration*			
Mean (SD)	0.11 (0.23)	0.10 (0.17)	0.11 [0.21]
Median [Min, Max]	0.05 [0.02, 3.13]	0.05 [0.02, 1.31]	0.05 [0.02, 3.13]
**Duration (hours)** ^*b^			
Mean (SD)	12.3 (9.8)	12.6 (9.9)	12.4 (9.8)
Median [Min, Max]	4.7 [2.0, 27.9]	4.7 [2.0, 28.0]	4.7 [2.0, 28.0]
**Dose (g/m**^**2**^**)** ^*b^			
Mean (SD)	4.2 (3.8)	4.1 (3.7)	4.2 (3.8)
Median [Min, Max]	3.5 [0.2, 12.1]	3.0 [0.2, 12.0]	3.5 [0.2, 12.1]
**Disease**			
Leukemia	59 (40.7%)	24 (38.1%)	83 (39.9%)
Lymphoma	74 (51.0%)	32 (50.8%)	106 (51.0%)
Sarcoma	10 (6.9%)	6 (9.5%)	16 (7.7%)
Missing	2 (1.4%)	1 (1.6%)	3 (1.4%)

^*^ Summarized based on each data point rather than individual level

^a^ Does not include observations below lower limit of quantification

^b^ Loading dose excluded from summary where applicable

Note: 1^st^ concentration typically measured at 24 hours, 2^nd^ concentration typically measured at 48 hours, 3^rd^ concentration typically measured at 72 hours

## Population PK model

The selected base model was a three-compartment model with proportional error, including random effects on all PK parameters and inter-occasion variability (IOV) on clearance. Allowing for correlation on the random effects did not improve the model. Table S1 in Supplemental Information provides the OFV, AIC, and BIC values for the base models that were considered. The three-compartment model is parameterized as clearance (CL, L/hour), volume of distribution in the main compartment (V1, L), intercompartmental clearance between the main compartment and the peripheral compartment (Q2, L/hour), volume of distribution in the peripheral compartment (V2, L), intercompartmental clearance between the main compartment and the secondary peripheral compartment (Q3, L/hour), and volume of distribution in the secondary peripheral compartment (V3, L).

Table 2 shows the parameter estimates of the base model, adjusted model, and final model for comparison of all PK parameters. The parameter estimates and coefficient of variation (%CV) from the base model were CL = 7.63 L/hour (29%), V1 = 17.88 L (43%), Q2 = 0.75 L/hour (32%), V2 = 5.32 L (37%), Q3 = 0.08 L/hour (59%), and V3 = 5.37 L (50%). BSA was selected for the adjusted model, as it consistently outperformed weight across all likelihood measures. Adding SCR and sex individually as covariates on CL significantly improved model fit, reducing the adjusted model OFV by 119.19 and 7.72, respectively. Including sex with the SCR model reduced the adjusted model OFV by 144.91 (from 119.19), with improved BIC and AIC. Thus, both were retained in the final model. The final model structure is as follows:

**Table 2:** Population PK modeling results

Base Model		BSA-adjusted Model		Final Model	
OFV = -2272.1 AIC = -2244.1 BIC = -2184.1		OFV = -2367.2 AIC = -2337.2 BIC = -2274.8		OFV = -2512.1 AIC = -2478.1 BIC = -2407.3	
Parameter	Est. (SE) [CI]	Parameter	Est. (SE) [CI]	Parameter	Est. (SE) [CI]
$\:\boldsymbol{C}\boldsymbol{L}=\:{\boldsymbol{\theta\:}}_{1\:}$		$\:\boldsymbol{C}\boldsymbol{L}=\:{\boldsymbol{\theta\:}}_{1\:}\times\:{\left(\boldsymbol{B}\boldsymbol{S}\boldsymbol{A}/1.97\right)}^{{\boldsymbol{\theta\:}}_{7}}$		$\:\boldsymbol{C}\boldsymbol{L}=\:{\boldsymbol{\theta\:}}_{1\:}$×${\left(\boldsymbol{B}\boldsymbol{S}\boldsymbol{A}/1.97\right)}^{{\boldsymbol{\theta\:}}_{7}}$×${\left(\boldsymbol{S}\boldsymbol{C}\boldsymbol{R}/68.08\right)}^{{\boldsymbol{\theta\:}}_{8}}\times\:\boldsymbol{e}\boldsymbol{x}\boldsymbol{p}\left({\boldsymbol{\theta\:}}_{9}\boldsymbol{*}\boldsymbol{I}\left(\boldsymbol{f}\boldsymbol{e}\boldsymbol{m}\boldsymbol{a}\boldsymbol{l}\boldsymbol{e}\right)\right)$	
θ_1_	7.63 (0.24) [7.18,8.11]	θ_1_	9.08 (0.25) [8.61,9.58]	θ_1_	9.41 (0.37) [8.72,10.16]
		θ_7_	0.46 (0.19) [0.21,0.97]	θ_7_	0.61 (0.14) [0.39,0.95]
				θ_8_	-0.56 (0.08) [-0.42,-0.74]
				θ_9_	0.01 (0.05) [-0.08,0.11]
$\:\boldsymbol{V}1=\:{\boldsymbol{\theta\:}}_{2\:}$		$\:\boldsymbol{V}1=\:{\boldsymbol{\theta\:}}_{2\:}\times\:{\left(\boldsymbol{B}\boldsymbol{S}\boldsymbol{A}/1.97\right)}^{1}$		$\:\boldsymbol{V}1=\:{\boldsymbol{\theta\:}}_{2\:}\times\:{\left(\boldsymbol{B}\boldsymbol{S}\boldsymbol{A}/1.97\right)}^{1}$	
θ_2_	17.88 (1.25) [15.6,20.5]	θ_2_	30.21 (1.63) [27.18,33.58]	θ_2_	30.67 (1.42) [28.01,33.57]
$\:\boldsymbol{Q}2=\:{\boldsymbol{\theta\:}}_{3\:}$		$\:\boldsymbol{Q}2=\:{\boldsymbol{\theta\:}}_{3\:}\times\:{\left(\boldsymbol{B}\boldsymbol{S}\boldsymbol{A}/1.97\right)}^{1}$		$\:\boldsymbol{Q}2=\:{\boldsymbol{\theta\:}}_{3\:}\times\:{\left(\boldsymbol{B}\boldsymbol{S}\boldsymbol{A}/1.97\right)}^{1}$	
θ_3_	0.75 (0.05) [0.66, 0.84]	θ_3_	0.71 (0.06) [0.60,0.85]	θ_3_	0.87 (0.07) [0.73,1.03]
$\:\boldsymbol{V}2=\:{\boldsymbol{\theta\:}}_{4\:}$		$\:\boldsymbol{V}2=\:{\boldsymbol{\theta\:}}_{4\:}\times\:{\left(\boldsymbol{B}\boldsymbol{S}\boldsymbol{A}/1.97\right)}^{1}$		$\:\boldsymbol{V}2=\:{\boldsymbol{\theta\:}}_{4\:}\times\:{\left(\boldsymbol{B}\boldsymbol{S}\boldsymbol{A}/1.97\right)}^{1}$	
θ_4_	5.32 (0.25) [4.86,5.83]	θ_4_	4.99 (0.27) [4.48,5.55]	θ_4_	5.62 (0.29) [5.09,6.21]
$\:\boldsymbol{Q}3=\:{\boldsymbol{\theta\:}}_{5\:}$		$\:\boldsymbol{Q}3=\:{\boldsymbol{\theta\:}}_{5\:}\times\:{\left(\boldsymbol{B}\boldsymbol{S}\boldsymbol{A}/1.97\right)}^{1}$		$\:\boldsymbol{Q}3=\:{\boldsymbol{\theta\:}}_{5\:}\times\:{\left(\boldsymbol{B}\boldsymbol{S}\boldsymbol{A}/1.97\right)}^{1}$	
θ_5_	0.08 (0.01) [0.07,0.10]	θ_5_	0.14 (0.01) [0.12,0.15]	θ_5_	0.14 (0.01) [0.11,0.17]
$\:\boldsymbol{V}3=\:{\boldsymbol{\theta\:}}_{6\:}$		$\:\boldsymbol{V}3=\:{\boldsymbol{\theta\:}}_{6\:}\times\:{\left(\boldsymbol{B}\boldsymbol{S}\boldsymbol{A}/1.97\right)}^{1}$		$\:\boldsymbol{V}3=\:{\boldsymbol{\theta\:}}_{6\:}\times\:{\left(\boldsymbol{B}\boldsymbol{S}\boldsymbol{A}/1.97\right)}^{1}\:$	
θ_6_	5.37 (0.50) [4.48,6.43]	θ_6_	12.05 (1.13) [10.05,14.46]	θ_6_	10.60 (2.43) [6.88,16.34]
ω_CL_ (%CV)	0.29 (0.03) [0.25,0.34]	ω_CL_ (%CV)	0.26 (0.02) [0.22,0.31]	ω_CL_ (%CV)	0.24 (0.02) [0.21,0.29]
ω_V1_ (%CV)	0.43 (0.05) [0.35,0.53]	ω_V1_ (%CV)	0.36 (0.04) [0.29,0.45]	ω_V1_ (%CV)	0.29 (0.03) [0.23,0.36]
ω_Q2_ (%CV)	0.32 (0.06) [0.23,0.45]	ω_Q2_ (%CV)	0.24 (0.11) [0.11,0.54]	ω_Q2_ (%CV)	0.38 (0.12) [0.21,0.69]
ω_V2_ (%CV)	0.37 (0.05) [0.28,0.48]	ω_V2_ (%CV)	0.26 (0.06) [0.17,0.41]	ω_V2_ (%CV)	0.30 (0.05) [0.21,0.42]
ω_Q3_ (%CV)	0.59 (0.06) [0.48,0.71]	ω_Q3_ (%CV)	0.45 (0.06) [0.36,0.57]	ω_Q3_ (%CV)	0.47 (0.05) [0.39,0.56]
ω_V3_ (%CV)	0.50 (0.12) [0.31, 0.79]	ω_V3_ (%CV)	0.43 (0.11) [0.26,0.68]	ω_V3_ (%CV)	0.34 (0.28) [0.10,1.15]
δ_CL_ (%CV)	0.17 (0.01) [0.15,0.18]	δ_CL_ (%CV)	0.16 (0.01) [0.15,0.18]	δ_CL_ (%CV)	0.14 (0.01) [0.13,0.15]
σ_proportional_ (%CV)	0.24 (0.01) [0.23,0.26]	σ_proportional_ (%CV)	0.25 (0.01) [0.24,0.26]	σ_proportional_ (%CV)	0.25 (0.01) [0.24,0.26]

Abbreviation: OFV, objective function value; AIC, Akaike information criterion; BIC, Bayesian information criterion; Est, point estimate; SE, standard error; CI, Wald 95% confidence interval; SCR, serum creatinine (µmol/L); BSA, body surface area (m^2^); I(female), indicator for female sex; θ_1_, θ_2_, θ_3_, θ_4_, θ_5_, θ_6_, θ_7_, θ_8_, and θ_9_, model parameters; η_i_^CL^, η_i_^V1^, η_i_^Q2^ , η_i_^V2^, η_i_^Q3^, and η_i_^V3^, individual random effects following a lognormal distribution with means zero and covariance matrix with variances along the diagonal; δ^CL^, intra-occasion variability on clearance; σ_proportional_, proportional residual error; %CV, percent coefficient of variation; CL, clearance (L/hour); V1, volume of distribution in the main compartment (L); Q2, intercompartmental clearance between the main compartment and the peripheral compartment (L/hour); V2, volume of distribution in the peripheral compartment (L); Q3, intercompartmental clearance between the main compartment and the secondary peripheral compartment (L/hour); V3, and volume of distribution in the secondary peripheral compartment (L)

$$\begin{aligned}\:{CL}_{ij}={\theta\:}_{1}\:\times\:\:{{(BSA}_{ij}/1.97)}^{{\theta\:}_{7}}\:\times\:\:{\left({SCR}_{ij}/68.08\right)}^{{\theta\:}_{8}}\\{\times\:\:{\text{ e}\mathrm{x}\mathrm{p}(\theta\:}_{9}\times I\left(female\right)}_{i})\:\times\:\:\mathrm{e}\mathrm{x}\mathrm{p}({{\eta\:}_{i}}^{CL}+\:{{\delta}_{i}}^{CL})\end{aligned},$$

$$\:{V1}_{ij}={\theta\:}_{2}\:\times\:\:{{(BSA}_{ij}/1.97)}^{1}\:\times\:\:\mathrm{e}\mathrm{x}\mathrm{p}\left({{\eta\:}_{i}}^{V1}\right),$$

$$\:{Q2}_{ij}={\theta\:}_{3}\:\times\:\:{{(BSA}_{ij}/1.97)}^{1}\:\times\:\:\mathrm{e}\mathrm{x}\mathrm{p}\left({{\eta\:}_{i}}^{Q2}\right),\:$$

$$\:{V2}_{ij}={\theta\:}_{4}\:\times\:\:{{(BSA}_{ij}/1.97)}^{1}\:\times\:\:\mathrm{e}\mathrm{x}\mathrm{p}\left({{\eta\:}_{i}}^{V2}\right),\:$$

$$\:{Q3}_{ij}={\theta\:}_{5}\:\times\:\:{{(BSA}_{ij}/1.97)}^{1}\:\times\:\:\mathrm{e}\mathrm{x}\mathrm{p}\left({{\eta\:}_{i}}^{Q3}\right),\:$$

$$\:{V3}_{ij}={\theta\:}_{6}\:\times\:\:{{(BSA}_{ij}/1.97)}^{1}\:\times\:\:\mathrm{e}\mathrm{x}\mathrm{p}\left({{\eta\:}_{i}}^{V3}\right),$$

where CL_ij_, V1_ij_, Q2_ij_, V2_ij_, Q3_ij_, and V3_ij_ are the individual specific PK parameters for individual *i* at time *j*, SCR_ij_ is the serum creatinine level in µmol/L for individual *i* at time *j*, BSA_ij_ is the body surface area in m^2^ for individual *i* at time *j*, $\:{I\left(female\right)}_{i}$ is an indicator for female sex, $\:{\theta\:}_{1},\:{\theta\:}_{2},\:{\theta\:}_{3},\:{\theta\:}_{4},\:{\theta\:}_{5},\:{\theta\:}_{6},{\theta\:}_{7},{\theta\:}_{8},{\text{and }\:\theta\:}_{9}$ are the model parameters, $\:{{\eta\:}_{i}}^{CL}$, $\:{{\eta\:}_{i}}^{V1}$, $\:{{\eta\:}_{i}}^{Q2}$, $\:{{\eta\:}_{i}}^{V2}$, $\:{{\eta\:}_{i}}^{Q3}$, and $\:{{\eta\:}_{i}}^{V3}\:$represent the individual random effects which follow a lognormal distribution with means zero and covariance matrix with variances along the diagonal, and $\:{{\delta}_{i}}^{CL}$ represents the IOV on CL. The parameter estimates and coefficient of variation (%CV) from the final model were CL = 9.41 L/hour (24%), V1 = 30.67 L (29%), Q2 = 0.87 L/hour (38%), V2 = 5.62 L (30%), Q3 = 0.14 L/hour (47%), and V3 = 10.60 L (34%). In general, the estimate of %CV was substantially reduced from the base model to the final model.

**Fig. 1:**
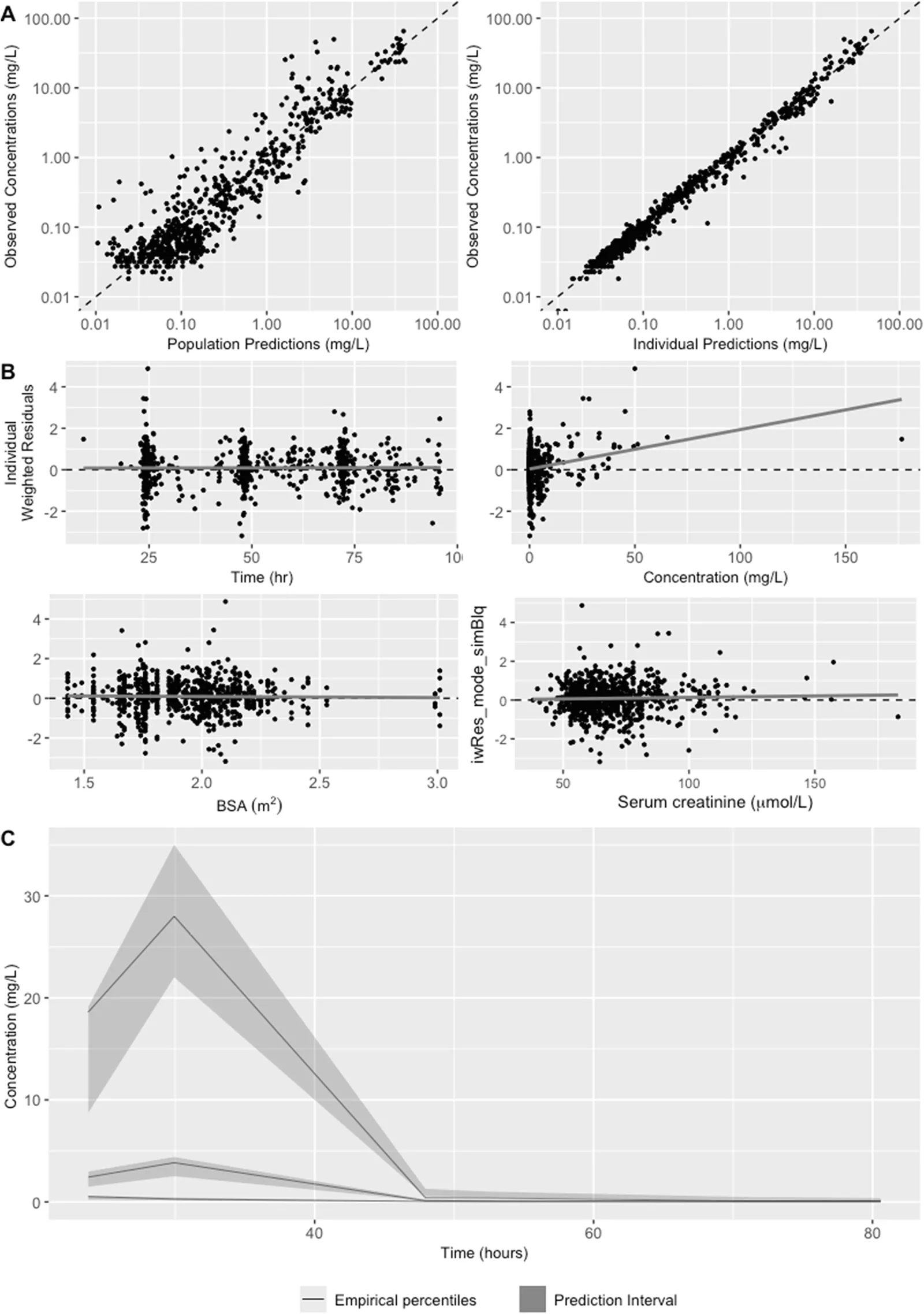
Model diagnostics for our final model. (**A**) Observed versus predicted plots for the population (left) and individual (right) predicted concentrations with the identity line (dashed). (**B**) Individual weighted residuals as a function of time (top left), observed concentrations (top right), body surface area (bottom left), and serum creatinine (bottom right) with a dashed line at zero residuals. (**C**) Visual predictive check with solid lines to represent empirical quartiles of concentration and shaded regions to represent the 10th, 50th, and 90th quantiles of each quartile as determined by simulations

Diagnostic plots for the final model based on the test dataset support a good overall fit (Fig. 1**)**. The observed versus population and individual prediction plots (Fig. 1A) are centered around the identify line, indicating good agreement. The individual weighted residuals (Fig. 1B) lack a clear trend over time, with residuals evenly scattered around zero. A slight increase in residuals is observed at higher concentrations and with higher SCR; however, this is likely driven by a small number of high-value outliers. The VPC (Fig. 1C) shows no evidence of systemic bias, particularly within 60 h – the key period for assessing early drug exposure.

## Model validation

Ten models, including the Taylor model, were selected for validation based on available covariates. Table 3 summarizes their structure and key characteristics. It includes 7 two-compartment models and 3 three-compartment models. All models incorporated weight or BSA and random effects on clearance; most included random effects on other PK parameters as well. IOV on clearance was included in 6 models. All models were fit on the full test cohort, except for the Hui models which were also fit to disease-specific subsets (ALL, OS).

**Table 3:** Summary of models that were evaluated, along with our final model

Model	Year	Number of Compartment	Cohort Location, dates Infusion length	Regressors	BSV, IOV, Residual error
Arshad et al. [33]	2021	3	Adult neutropenic patients (neutrophils < 500 /mm3) with hematological malignancies or solid tumors Germany, January 2005 through February 2018 4–24 h IV	CL: SCR*, Age*, Sex*, BSA* V1: None Q2: None V2: None Q3: None V3: None	BSV: CL, V1 IOV: CL Combined error
Beechinor et al. [34]	2019	2	Infants with ALL US, 2001 through 2006 24 h IV	CL: Weight^0.75^ V1: Weight^1^ Q: Weight^0.75^ V2: Weight^1^	BSV: CL and V1 IOV: CL Proportional error
Gallais et al. [35]	2020	2	Adult patients with ALL or NHL France, January 2012 through September 2018 Long-term and short-term IV with occasional loading dose	CL: Age^*^ V1: None Q^**a**^: None V2: Weight^*^	BSV: CL and V2 IOV: CL Proportional error
Gao et al. [36]	2021	3	Pediatric patients with ALL Shanghai, January 2014 through December 2019 24 h IV	CL: Weight^0.75^, SCR^*^ V1: Weight^1^ Q2: Weight^0.75^ V2: Weight^1^ Q3: Weight^0.75^ V3: Weight^1^	BSV: CL and Q1 Additive error
Hui et al.^b^ ALL model [37]	2019	2	Pediatric patients with ALL Hong Kong, January 2004 through August 2014 24 h IV	CL: CrCl^*^, BSA^*^ V1: BSA^*^ Q: Age V2: None	BSV: CL, V2 IOV: CL Proportional error
Hui et al. OS model [37]	2019	2	Pediatric patients with Osteosarcoma Hong Kong, January 2004 through August 2014 6 h IV	CL: Height^*^, CrCl^*^, Dose^*^ V1: Height^*^ Q: Weight^*^ V2: Age^*^	BSV: CL, V1, Q, V2 IOV: CL Proportional error
Maximova et al. [38]	2025	2	Pediatric patients with ALL 30 min IV loading dose plus 23.5 h IV	CL: SCR^*^ V1: Weight^1^ Q: None V2: None	BSV: CL, Q IOV: CL Proportional error
Medellin-Garibay et al. [39]	2019	2	Pediatric patients with ALL Mexico, August 2016 through December 2018. 24 h IV with loading dose	CL: BSA^*^ V1: Weight^1^ Q: None V2: None	BSV: CL, V1, and V2 Proportional error
Mei et al. [40]	2018	2	Pediatric and adult patients China, September 2016 through August 2017 Short-term IV	CL: SCR^*^, BSA^†*^ V1: Age^*^ Q: None V2: None	BSV: CL, V1, Q, V2 Proportional error
Olivo et al. [41]	2024	2	Pediatric patients with Osteosarcoma Brazil, January 2015 through March 2023 4 h IV	CL: SCR^*^ V1: BSA^*^ Q: None V2: None	BSV: CL, V1 IOV: CL
Taylor et al. [3]	2020	3	Pediatric patients with Philadelphia chromosome-negative ALL Nordic countries, January 2002 through December 2014 24 h IV	CL: BSA^1^, SCR^*^ V1: BSA^1^ Q2: BSA^1^ V2: BSA^1^ Q3: BSA^1^ V3: BSA^1^	BSV: CL, V2, Q3, V3 Proportional error
Our final model	2025	3	Adult patients with malignancies US, November 2017 through December 2022 Long-term and short-term IV with occasional loading dose	CL: BSA^*^, SCR^*^, Sex^*^ V1: BSA^1^ Q2: BSA^1^ V2: BSA^1^ Q3: BSA^1^ V3: BSA^1^	BSV: CL, V1, Q2, V2, Q3, V3 IOV: Cl Proportional error

^**a**^ Q is used as a fixed value

^**b**^ Hui et al. reported separate population pharmacokinetic models for acute lymphoblastic leukemia (ALL) and osteosarcoma (OS) cohorts; these are referred to as “Hui ALL” and “Hui osteosarcoma,” respectively.

^*^ Regression coefficient was estimated without being fixed

Abbreviation: BSV, between-subject variability; IOV, inter-occasion variability; CL, clearance from the main compartment; V1, volume of distribution in the main compartment; Q2, intercompartmental clearance between the main compartment and the peripheral compartment; V2, volume of distribution in the peripheral compartment; Q3, volume of distribution in the second peripheral compartment; V3, intercompartmental clearance between the main compartment and the second peripheral compartment; Q, intercompartmental clearance between the main compartment and the peripheral compartment for a two-compartment model; BSA, body surface area; SCR, serum creatinine; IV, intravenous infusion; ALL, acute lymphoblastic leukemia; NHL, non-Hodgkin lymphoma.

CrCl, creatinine clearance defined by the Schwartz formula of 0.413× height/SCR.

BSA^†^, defined by 1.05 + (weight – 30) $\:\times\:\:$ 0.02 for weight > 30 kg or 0.035 $\:\times\:$ weight + 0.1 for weight $\:\le\:$ 30 kg

Across all models, MAPE exceeded MPE, indicating imprecision (Table 4). Our model performed similarly to the Gallais model across RMSE, MAPE, F20, and F30 metrics, with both achieving the highest F20 and F30 values among all candidates. However, neither met predefined performance criteria: MPE ≤ 20%, MAPE ≤ 30%, F20 ≥ 35%, and F30 ≥ 50% [12]. Among models with positive MPE, only our model combined low RMSE with high F20 and F30. Although the Arshad and Gao models had positive MPE, both had substantially higher RMSE (2–3 mg/L higher than other candidate models). In contrast, the Gallais, Hui ALL, Olivo, and Taylor models had negative MPE, indicating under-prediction. The Taylor model’s performance was not clearly superior or inferior. The observed versus population-predicted plots confirm poor fit for the Arshad, Gao, Hui OS, Maximova, Medellin-Garibay, and Mei models (Figure S1 in Supplemental Information).

**Table 4:** Predictive performance of all evaluated models

Model	RMSE	MPE	MAPE	F20	F30
Arshad	9.24	12.57	44.73	24.37	34.96
Beechinor	4.57	1.73	53.73	17.55	27.72
Gallais	6.41	-23.66	40.90	24.79	36.91
Gao	9.69	725.26	725.26	6.41	8.64
Hui^a^ ALL on ALL cohort	4.47	-22.62	46.61	21.37	32.05
Hui ALL on full cohort	6.04	-29.77	46.79	21.73	31.62
Hui OS on OS cohort	4.90	120.66	120.66	11.61	15.48
Hui OS on full cohort	14.81	844.80	844.80	3.06	4.04
Maximova	9.06	-49.24	92.01	7.10	10.03
Medellin-Garibay	6.23	-71.69	74.47	11.98	16.57
Mei	6.62	-99.93	99.95	4.60	6.96
Olivo	6.64	-21.59	48.53	19.36	31.75
Taylor	6.74	-26.07	47.21	21.73	30.36
Our final model	5.91	10.61	46.88	24.51	35.52

^**a**^ Hui et al. reported separate population pharmacokinetic models for acute lymphoblastic leukemia (ALL) and osteosarcoma (OS) cohorts; these are referred to as “Hui ALL” and “Hui OS,” respectively.

Abbreviation: RMSE, root mean squared error; MPE, median percent prediction error; MAPE, median absolute percent prediction error; F20, proportion of percent prediction errors between − 20% and 20%; F30, proportion of percent prediction errors between − 30% and 30%

Based on overall performance, the Beechinor, Gallais, Hui ALL, Olivo, and Taylor models were selected for refitting and Bayesian forecasting. The Hui ALL model performed relatively consistent between the full cohort and indication-specific subset, so we proceeded with only the full cohort to maintain consistency with other evaluated models. The Arshad model was excluded for high RMSE and low precision at high concentrations. The Gao, Hui OS, Maximova, Medellin-Garibay, and Mei models were excluded for high bias (i.e., high MPE and MAPE) and poor predictive performance (i.e., low F20 and F30). After refitting, our model demonstrated among the best overall performance across MPE, MAPE, F20, and F30 (Table S2 in Supplemental Information). Differences between the top-performing models were minimal (slightly lower MAPE for the Olivo model and slightly higher F20 for the Hui ALL model). All models had better RMSE compared to our model. Our model also yielded the lowest OFV, AIC, and BIC values (Table S3 in Supplemental Information).

### Bayesian forecasting

**Fig. 2:**
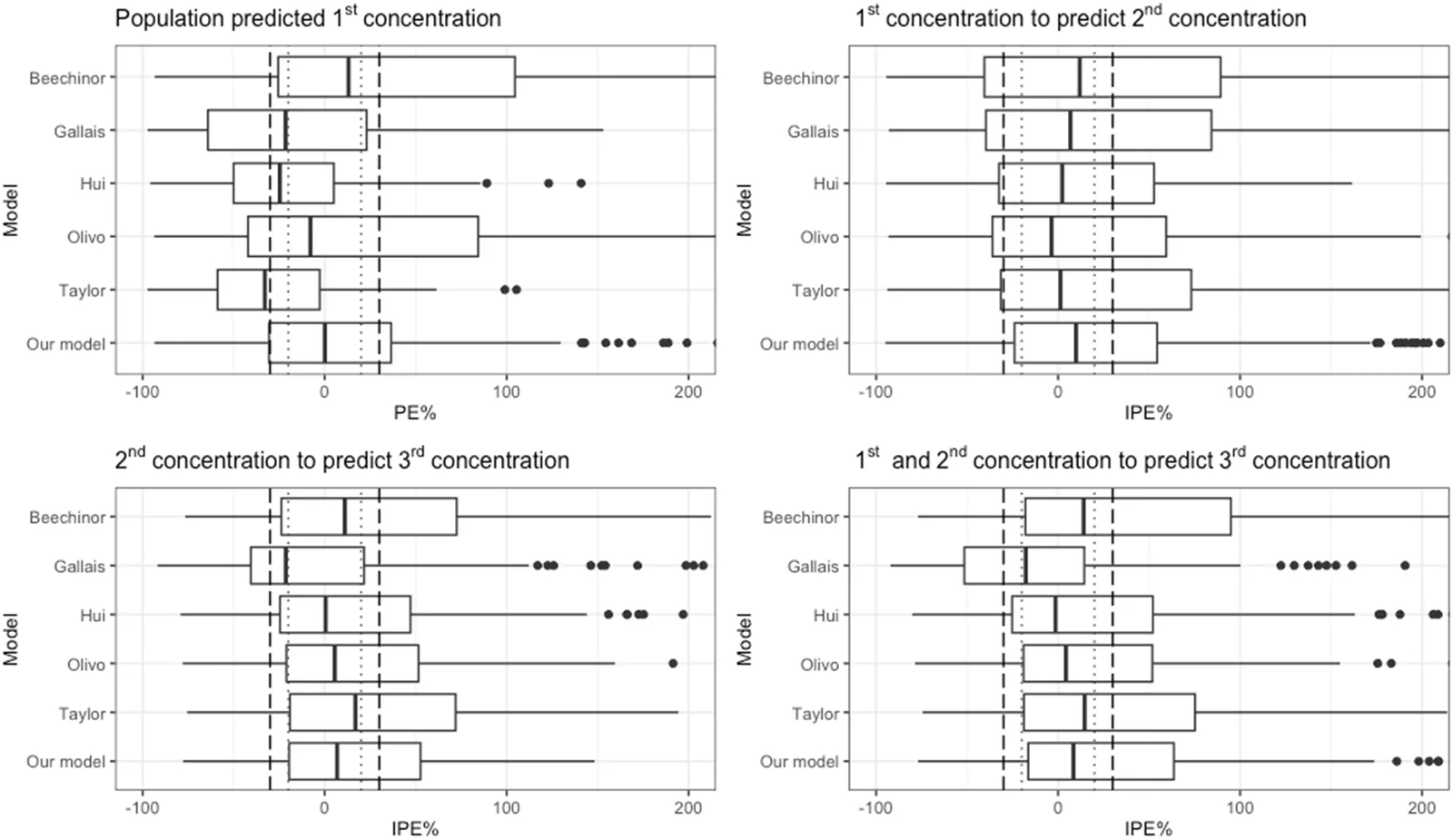
Boxplots of PE% and IPE%. Data limited to the range of -100% to 200%. The four panels represent: the first population-predicted concentration (top left); the Bayesian forecast of the second concentration using the first concentration as prior information (top right); the Bayesian forecast of the third concentration using the second concentration as prior information (bottom left); and the Bayesian forecast of the third concentration using the first and second concentrations as prior information (bottom right). Note: All panels exclude outliers above 200%. Grey dotted lines at -20% and 20% visualize the IF20 and black dashed lines at -30% and 30% visualize the IF30. Abbreviation: PE%, percent prediction error; IPE%, individual percent prediction error; F20, proportion of percent prediction errors between − 20% and 20%; F30, proportion of percent prediction errors between − 30% and 30%

Bayesian forecasting improved prediction accuracy. Table 5 summarizes the predictive performance, and Fig. 2 presents the distributions of PE% and IPE%, with benchmarks at 20% and 30%. Figure 2 is zoomed to the central range of the boxplots; the figure showing the full range, including outliers, is provided in Figure S2. Predictions were compared at the first, second, and third concentrations (typically drawn 24, 48, and 72 h post-dose, respectively).

**Table 5:** Predictive performance of Bayesian forecasting on re-fit models and our final model

Model	IRMSE	MIPE	MAIPE	IF20	IF30
Beechinor					
Predict 1st^**a**^	8.49	13.10	45.58	23.90	34.63
1st predict 2nd	0.93	11.88	53.96	20.73	30.57
2nd predict 3rd	0.09	11.57	37.90	26.74	39.53
1st and 2nd predict 3rd	0.12	15.06	36.88	30.81	41.86
Gallais					
Predict 1st^**a**^	11.94	-21.46	43.83	22.93	37.56
1st predict 2nd	0.76	6.76	56.20	20.72	29.53
2nd predict 3rd	0.11	-21.20	38.11	21.51	36.63
1st and 2nd predict 3rd	0.12	-17.70	41.83	27.91	40.70
Hui ALL model					
Predict 1st^**a**^	11.26	-24.65	34.89	29.76	42.93
1st predict 2nd	0.82	2.26	37.41	27.98	39.90
2nd predict 3rd	0.09	0.70	29.78	24.88	50.00
1st and 2nd predict 3rd	0.17	-1.29	33.34	31.98	47.09
Olivo					
Predict 1st^**a**^	12.39	-7.82	50.08	16.10	31.71
1st predict 2nd	0.75	-3.63	40.54	22.28	36.79
2nd predict 3rd	0.08	5.71	31.10	36.05	49.42
1st and 2nd predict 3rd	0.14	4.31	30.91	35.47	48.84
Taylor					
Predict 1st^**a**^	12.57	-32.85	42.07	26.83	37.56
1st predict 2nd	0.71	1.26	44.07	22.80	36.79
2nd predict 3rd	0.10	17.23	36.49	26.74	41.86
1st and 2nd predict 3rd	0.11	14.64	37.74	31.40	41.28
Our final model					
Predict 1st^**a**^	10.99	0.10	33.81	31.22	44.39
1st predict 2nd	0.98	9.86	34.59	23.83	44.04
2nd predict 3rd	0.10	8.16	32.31	33.14	47.09
1st and 2nd predict 3rd	0.14	8.62	30.08	33.14	50.00

^**a**^ The Predict 1st performance metrics are based on population prediction as the model cannot be updated before the first concentration is observed. Therefore, the individual prediction for the first concentration is the same as the population prediction.

Note: The first concentrations are typically measured at 24 hours, the second at 48 hours, and the third at 72 hours.

Abbreviation: IRMSE, individual root mean squared error; MIPE, median individual percent prediction error; MAIPE, median absolute individual percent prediction error; IF20, proportion of individual percent prediction errors between − 20% and 20%; IF30, proportion of individual percent prediction errors between − 30% and 30%; RMSE, root mean squared error; MPE, median percent prediction error; MAPE, median absolute percent prediction error; F20, proportion of percent prediction errors between − 20% and 20%; F30, proportion of percent prediction errors between − 30% and 30%; ALL, acute lymphoblastic leukemia

At the first concentration, our model outperforms the others across most metrics (i.e., MPE, MAPE, F20, and F30). Its MPE is close to zero, as reflected in the PE% distribution, which is tightly centered around 0.10, resulting in F30 of 44.39%. The next best MPE is 13.10 from the Beechinor model, a value that differs substantially from ours, whereas the Gallais, Hui ALL, Olivo, and Taylor models show large negative MPE values, indicating substantial under-prediction.

When predicting the second concentration using the first as prior information, performance differences between models were modest. The Taylor model achieved the lowest MIPE, while the Hui ALL model demonstrated the best overall balance of bias and precision with low MIPE, the lowest MAIPE, and the highest IF20 and IF30. While all models show similar IPE% distribution, our model tends to over-predict (Fig. 2).

Predicting the third concentration showed similar patterns across forecasting scenarios. Including both the first and the second concentrations provided only modest gains compared with only including the second concentration. Our model achieved the highest IF30 when both prior concentrations were used, but the Hui ALL and Olivo models demonstrated comparable results and the Olivo model showed superior IF20. Overall, at the third concentration, the Hui ALL and Olivo models and our model maintained strong overall performance.

## Discussion

We evaluated various HD-MTX popPK models, including a newly developed model, by assessing their predictive performance on a cohort of patients at VUMC. Our objective was to identify the most suitable model for predicting MTX levels after HD-MTX administration in VUMC adult patients, enabling optimization of therapy and early detection of delayed clearance and avoidance of toxicity.

We developed a three-compartment model with BSA, SCR, and sex as covariates. The inclusion of sex was unexpected. While BSA and SCR are well-established factors that may influence clearance, sex is not typically considered a key predictor. However, retention of sex in the final model results in a substantial reduction in OFV (25.7 units) and lower AIC (23.7 units) and BIC (19.5 units). The model including sex also demonstrated improved RMSE and MPE, while maintaining comparable precision (i.e., MAPE, F20, and F30) relative to the model without sex (Table **S4** in Supplemental Information). Based on these improvements in goodness-of-fit and accuracy, sex was retained in the final model. This effect may be partially explained by differences in BSA between males and females, contributing to variability in PK parameters. Additionally, differences in body composition, particularly body fat distribution, may influence clearance. Notably, to address sex-based difference, Seydoux et al. proposed a sex-adjusted BSA [19].

Our final model showed comparable or improved population-level predictive performance relative to external models. Although observed versus predicted plots revealed some under-prediction, these cases were associated with treatment cycles involving significant changes in SCR or weight—key predictors of delayed MTX clearance.

Similar performance metrics across models highlight the need for more nuanced evaluation criteria. Threshold-based metrics such as F20 and F30 provide insight beyond traditional central tendency measures and can improve model comparison and validation.

A challenge in using Bayesian forecasting for our dataset is that dosing only occurs at the beginning of each cycle. Consequently, the first concentration (typically the highest and most clinically critical) lacks prior data to inform prediction. Later concentrations, which can incorporate prior data, often occur at low MTX levels where small absolute differences in observed and predicted values may inflate PE%.

Our model provided the most accurate population-level predictions for the first measured concentration (peak), which is clinically crucial, as minimizing toxicity is a primary goal of TDM. Although the Hui ALL model demonstrated the highest precision and accuracy at the second concentration via Bayesian forecasting, our model had less under-prediction at this time and achieved comparable IF20 and IF30 (Fig. 2; Table 5). Overall, the Beechinor and Gallais models performed worse across all time points, possibly due to differences in model structure. The Beechinor model, for instance, employs a simpler two-compartment model, which may reduce its ability to accurately capture peak concentrations and contribute to greater variability in prediction.

At the third measurement, differences in performance across models were modest, reflecting a broader trade-off in predictive performance across time points. Clear shifts in PE% distributions are observed between the first and the second concentrations (Fig. 2**)**. Our model predicts the first concentration well, tends to over-predict the second, and performs reasonably at the third. In contrast, external models, particularly the Hui ALL and Olivo models, demonstrate improved accuracy at later time points once concentration data are incorporated. Notably, both models include IOV on clearance, similar to our model, which may contribute to their improvements by better accounting for within-patient variability across dosing occasions.

These findings highlight that performance at one time point does not necessarily translate to other time points. HD-MTX monitoring decisions should account for this trade-off. While our model should serve as the primary basis for HD-MTX TDM at VUMC, the Hui ALL model (and the Olivo model) may provide complementary value at later time points (e.g., 48 h and 72 h) due to their improved accuracy.

One explanation for our model’s superior performance at the first concentration lies in the population used for model development. Many comparator models (Beechinor, Gao, Medellin-Garibey, Hui, Maximova, Olivo, and Taylor) were developed using pediatric data. These models underperform when no prior information is available (Table 5), suggesting that models developed in pediatric populations may be less suitable for population-level prediction in adults. Although Arshad et al. developed their model using adult data, their cohort was limited to neutropenic patients, representing a higher risk subgroup and limiting generalizability. Collectively, these findings underscore the need for popPK models specifically developed in adult patients receiving HD-MTX, particularly to enable accurate prediction at early time points when prior concentration data are unavailable.

Our study has several limitations. The dataset included 208 individuals, with substantial variability in treatment cycles per patient (range 1–12 cycles; median 3). Patients contributing more cycles may have disproportionately influenced the model. A larger sample size including multiple sites would improve the generalizability and the reliability of parameter estimates, especially for a three-compartment model. Although EHR data can contain errors or inconsistencies, some of which we detected during data quality checks, data obtained from structured forms generally have low error rate and are unlikely to meaningfully affect model performance. The use of EHR data with an emphasis on readily available bedside measurements restricted the covariates included in our model and which models could be evaluated during external validation. Several variables used in external models were unavailable or frequently missing in our dataset, including albumin [20–23], alanine aminotransferase [24], hematocrit [25], hemoglobin [26], and eGFR [27]. While these markers of hepatic and renal function are associated with HD-MTX toxicity risk [28], they were not consistently available at relevant time points. We were also unable to include an updated version of the Taylor model which contains Down syndrome diagnosis, severe hypoalbuminemia, and pleural effusion as covariates [29]. Similarly, genetic information [20, 30] and concurrent medications [31, 32], included in more recent models, were not available in our dataset.

Future work will focus on developing a TDM tool to support personalized HD-MTX therapy for adult patients. The tool will be designed for clinician use to more accurately predict when a patient will need glucarpidase. The decision-making process will be tailored to the measurement time of interest, with the tool incorporating our final model along with the Taylor and Hui ALL models. The generated time-concentration profile will help pharmacists and hematology/oncology physicians identify patients at risk of delayed clearance and guide timely intervention. The tool will include a visual aid comparing predictions against the consensus glucarpidase guideline thresholds, as well as a downloadable table of predicted concentrations at 12-hour intervals (.csv format).

## Supplementary Information

Below is the link to the electronic supplementary material.

Supplementary Material 1

## Data Availability

The data supporting the findings of this study are not publicly available due to restrictions imposed by the Institutional Review Board and the informed consent provided by study participants. Data may be available from the corresponding author upon reasonable request and with approval from the appropriate ethics committee.

## References

[1] Jolivet J, Cowan KH, Curt GA et al (1983) The pharmacology and clinical use of methotrexate. N Engl J Med 309:1094–1104. 10.1056/NEJM198311033091805 6353235

[2] Ramsey LB, Bruun GH, Yang W et al (2012) Rare versus common variants in pharmacogenetics: SLCO1B1 variation and methotrexate disposition. Genome Res 22:1–8. 10.1101/gr.129668.111 PMC3246196 22147369

[3] Taylor ZL, Mizuno T, Punt NC et al (2020) MTXPK.org: A Clinical Decision Support Tool Evaluating High-Dose Methotrexate Pharmacokinetics to Inform Post-Infusion Care and Use of Glucarpidase. Clin Pharmacol Ther 108:635–643. 10.1002/cpt.1957 PMC7484917 32558929

[4] (2022) ACCP Abstract Booklet. Clin Pharmacol Drug Dev 11:1–112. 10.1002/cpdd.1151 36089757

[5] Yang Y, Wang C, Chen Y et al (2023) External evaluation and systematic review of population pharmacokinetic models for high-dose methotrexate in cancer patients. Eur J Pharm Sci Off J Eur Fed Pharm Sci 186:106416. 10.1016/j.ejps.2023.106416 37119861

[6] Yang Y, Liu Z, Chen J et al (2023) Factors influencing methotrexate pharmacokinetics highlight the need for individualized dose adjustment: a systematic review. Eur J Clin Pharmacol 80:1–27. 10.1007/s00228-023-03579-0 37934204

[7] Pesenti G, Foppoli M, Manca D (2021) A minimal physiologically based pharmacokinetic model for high-dose methotrexate. Cancer Chemother Pharmacol 88:595–606. 10.1007/s00280-021-04305-2 PMC8367929 34120234

[8] Wei S, Zhang S, Wang D et al (2025) Population pharmacokinetics of high-dose methotrexate in patients with primary central nervous system lymphoma. Front Pharmacol 16:1578033. 10.3389/fphar.2025.1578033 PMC12127635 40458790

[9] Widemann BC, Adamson PC (2006) Understanding and managing methotrexate nephrotoxicity. Oncologist 11:694–703. 10.1634/theoncologist.11-6-694 16794248

[10] Howard SC, McCormick J, Pui C-H et al (2016) Preventing and Managing Toxicities of High-Dose Methotrexate. Oncologist 21:1471–1482. 10.1634/theoncologist.2015-0164 PMC5153332 27496039

[11] Lopez-Lopez E, Martin-Guerrero I, Ballesteros J et al (2011) Polymorphisms of the SLCO1B1 gene predict methotrexate-related toxicity in childhood acute lymphoblastic leukemia. Pediatr Blood Cancer 57:612–619. 10.1002/pbc.23074 21387541

[12] Komenkul V, Sukarnjanaset W, Komolmit P, Wattanavijitkul T (2024) External validation of population pharmacokinetic models of tacrolimus in Thai adult liver transplant recipients. Eur J Clin Pharmacol 80:1229–1240. 10.1007/s00228-024-03692-8 38695888

[13] Choi L, Beck C, McNeer E et al (2020) Development of a System for Postmarketing Population Pharmacokinetic and Pharmacodynamic Studies Using Real-World Data From Electronic Health Records Clin Pharmacol Ther 107:934–943. 10.1002/cpt.1787 PMC7093250 31957870

[14] James NT, Breeyear JH, Caprioli R et al (2022) Population pharmacokinetic analysis of dexmedetomidine in children using real-world data from electronic health records and remnant specimens. Br J Clin Pharmacol 88:2885–2898. 10.1111/bcp.15194 PMC9106818 34957589

[15] Ramsey LB, Balis FM, O’Brien MM et al (2018) Consensus Guideline for Use of Glucarpidase in Patients with High-Dose Methotrexate Induced Acute Kidney Injury and Delayed Methotrexate Clearance. Oncologist 23:52–61. 10.1634/theoncologist.2017-0243 PMC5759822 29079637

[16] MonolixSuite Documentation https://monolixsuite.slp-software.com/?l=en. Accessed 11 July 2025

[17] R: The R Project for Statistical Computing. https://www.r-project.org/. Accessed 11 July 2025

[18] NONMEM | Nonlinear Mixed Effects Modelling | ICON plc. https://www.iconplc.com/solutions/technologies/nonmem. Accessed 11 July 2025

[19] Seydoux C, Briki M, Wagner AD et al (2025) Importance of Sex-Dependent Differences for Dosing Selection and Optimization of Chemotherapeutic Drugs. Chemotherapy 70:92–101. 10.1159/000542461 PMC12101808 39510060

[20] Mao J-J, Jiao Z, Yun H-Y et al (2018) External evaluation of population pharmacokinetic models for ciclosporin in adult renal transplant recipients. Br J Clin Pharmacol 84:153–171. 10.1111/bcp.13431 PMC5736841 28891596

[21] Pai MP, Debacker KC, Derstine B et al (2020) Comparison of Body Size, Morphomics, and Kidney Function as Covariates of High-Dose Methotrexate Clearance in Obese Adults with Primary Central Nervous System Lymphoma. Pharmacother J Hum Pharmacol Drug Ther 40:308–319. 10.1002/phar.2379 PMC8855476 32090349

[22] Yin A, de Groot FA, Guchelaar H-J et al (2025) Population Pharmacokinetic and Toxicity Analysis of High-Dose Methotrexate in Patients with Central Nervous System Lymphoma. Clin Pharmacokinet 64:79–91. 10.1007/s40262-024-01452-6 39625585

[23] Zhao J, Wu R, Zhang S et al (2025) Population pharmacokinetic model of high-dose methotrexate in Chinese patients with intracranial germ cell tumors. Front Pharmacol 16:1548203. 10.3389/fphar.2025.1548203 PMC12081364 40385482

[24] Kawakatsu S, Nikanjam M, Lin M et al (2019) Population pharmacokinetic analysis of high-dose methotrexate in pediatric and adult oncology patients. Cancer Chemother Pharmacol 84:1339–1348. 10.1007/s00280-019-03966-4 31586225

[25] Nader A, Zahran N, Alshammaa A et al (2017) Population Pharmacokinetics of Intravenous Methotrexate in Patients with Hematological Malignancies: Utilization of Routine Clinical Monitoring Parameters. Eur J Drug Metab Pharmacokinet 42:221–228. 10.1007/s13318-016-0338-1 27059845

[26] Škorić B, Jovanović M, Kuzmanović M et al (2024) Understanding hemoglobin contribution to high-dose methotrexate disposition-population pharmacokinetics in pediatric patients with hematological malignancies. Eur J Clin Pharmacol 80:697–705. 10.1007/s00228-024-03642-4 38347227

[27] Panetta JC, Roberts JK, Huang J et al (2020) Pharmacokinetic basis for dosing high-dose methotrexate in infants and young children with malignant brain tumours. Br J Clin Pharmacol 86:362–371. 10.1111/bcp.14160 PMC7015755 31657864

[28] Howard SC, McCormick J, Pui C-H et al (2016) Preventing and Managing Toxicities of High-Dose Methotrexate. Oncologist 21:1471–1482. 10.1634/theoncologist.2015-0164 PMC5153332 27496039

[29] Taylor ZL, Miller TP, Poweleit EA et al (2023) Clinical covariates that improve the description of high dose methotrexate pharmacokinetics in a diverse population to inform MTXPK.org. Clin Transl Sci 16:2130–2143. 10.1111/cts.13600 PMC10651646 37503924

[30] Zhan M et al (2022) Sun,Yiqi, Zhou, Fang, Population pharmacokinetics of methotrexate in paediatric patients with acute lymphoblastic leukaemia and malignant lymphoma. Xenobiotica 52:265–273. 10.1080/00498254.2022.2069060 35446233

[31] Zhang Y, Qi X, Huang X et al (2024) An interactive dose optimizer based on population pharmacokinetic study to guide dosing of methotrexate in Chinese patients with osteosarcoma. Cancer Chemother Pharmacol 94:733–745. 10.1007/s00280-024-04708-x 39180550

[32] Chen X, Li J, Yu L et al (2024) High-dose methotrexate pharmacokinetics and its impact on prognosis of paediatric acute lymphoblastic leukaemia patients: A population pharmacokinetic study. Br J Haematol 204:1354–1366. 10.1111/bjh.19365 38432257

[33] Arshad U, Taubert M, Seeger-Nukpezah T et al (2021) Evaluation of body-surface-area adjusted dosing of high-dose methotrexate by population pharmacokinetics in a large cohort of cancer patients. BMC Cancer 21:1–10. 10.1186/s12885-021-08443-x PMC8214796 34147089

[34] Beechinor RJ, Thompson PA, Hwang MF et al (2019) The population pharmacokinetics of high-dose methotrexate in infants with acute lymphoblastic leukemia highlight the need for bedside individualized dose adjustment: a report from the Children’s Oncology Group. Clin Pharmacokinet 58:899. 10.1007/s40262-018-00734-0 PMC6658326 30810947

[35] Gallais F, Oberic L, Faguer S et al (2021) Body Surface Area Dosing of High-Dose Methotrexate Should Be Reconsidered, Particularly in Overweight, Adult Patients. Ther Drug Monit 43:408. 10.1097/FTD.0000000000000813 32925658

[36] Gao X, Qian X-W, Zhu X-H et al (2021) Population Pharmacokinetics of High-Dose Methotrexate in Chinese Pediatric Patients With Acute Lymphoblastic Leukemia. Front Pharmacol 12. 10.3389/fphar.2021.701452 PMC8313761 34326772

[37] Hui KH, Chu HM, Fong PS et al (2019) Population Pharmacokinetic Study and Individual Dose Adjustments of High-Dose Methotrexate in Chinese Pediatric Patients With Acute Lymphoblastic Leukemia or Osteosarcoma. J Clin Pharmacol 59:566–577. 10.1002/jcph.1349 30556906

[38] Maximova N, Calabrò PF, Cangialosi A et al (2025) Therapeutic Drug Monitoring, Population Pharmacokinetics Models, and External Validation of High-Dose Methotrexate in Pediatric Acute Lymphoblastic Leukemia. Chemotherapy 70:109–118. 10.1159/000543181 39694022

[39] Medellin-Garibay SE, Hernández-Villa N, Correa-González LC et al (2020) Population pharmacokinetics of methotrexate in Mexican pediatric patients with acute lymphoblastic leukemia. Cancer Chemother Pharmacol 85:21–31. 10.1007/s00280-019-03977-1 31673826

[40] Mei S, Li X, Jiang X et al (2018) Population Pharmacokinetics of High-Dose Methotrexate in Patients With Primary Central Nervous System Lymphoma. J Pharm Sci 107:1454–1460. 10.1016/j.xphs.2018.01.004 29331383

[41] Olivo LB, de Oliveira Henz P, Wermann S et al (2024) Anticipating Leucovorin Rescue Therapy in Patients with Osteosarcoma through Methotrexate Population Pharmacokinetic Model. Pharmaceutics 16:1180. 10.3390/pharmaceutics16091180 PMC11434990 39339216

