# Supplementary material for: Development and validation of high-dose methotrexate population pharmacokinetic models to inform clinical decisions on dosing
Source: Eur J Clin Pharmacol. 2026 May 23;82(6):156. doi: 10.1007/s00228-026-04080-0 (PMC13197298; doi:10.1007/s00228-026-04080-0)
Supplement: Supplementary file 1 — Supplementary Material 1 [file 228_2026_4080_MOESM1_ESM.docx]

*European Journal of Clinical Pharmacology*

**Development and Validation of High-Dose Methotrexate Population Pharmacokinetic Models to Inform Clinical Decisions on Dosing**

Marisa H. Blackman, MS^1^, Bradley Yelvington, PharmD^2^, Cole Beck, BS^1^,

Manuel Cortez, PharmD^3^, Leena Choi, PhD^1^

^1^Department of Biostatistics, Vanderbilt University Medical Center, Nashville, TN, USA
^2^Department of Oncology Pharmacy, Vanderbilt University Medical Center, Nashville, TN, USA

^3^Department of Pharmacy, Rush MD Anderson Cancer Center, Chicago, IL, USA

Corresponding author: Leena Choi [leena.choi@vumc.org](mailto:leena.choi@vumc.org)

**Supplemental Information**

**Table S1: Comparison of base models**

| **NCPT** | **Error Model** | **OFV** | **AIC** | **BIC** |
| --- | --- | --- | --- | --- |
| 1 | Additive | 5375.81 | 5385.81 | 5410.81 |
| 1 | Proportional | 673.81 | 685.81 | 710.81 |
| 1 | Combined | 29.64 | 43.64 | 72.80 |
| 2 | Additive | 4595.45 | 4614.45 | 4345.10 |
| 2 | Proportional | -2156.50 | -2136.50 | -2094.85 |
| 2 | Combined | -2150.17 | -2128.17 | -2082.35 |
| 3 | Additive | 2344.78 | 2372.78 | 2431.10 |
| **3** | **Proportional** | **-2272.14** | **-2244.14** | **-2185.82** |
| 3 | Combined | -2282.54 ^*^ | -2222.54 ^*^ | -2097.58 ^*^ |

**^*^** The 3-compartment model with combined error model had estimation issues. The additive error component was estimated at 0, which supports a proportional error model.

Abbreviation: NCPT, number of compartments; CL, clearance

**Figure S1:** **Observed versus population predicted concentrations for the external models and our final model.**

**
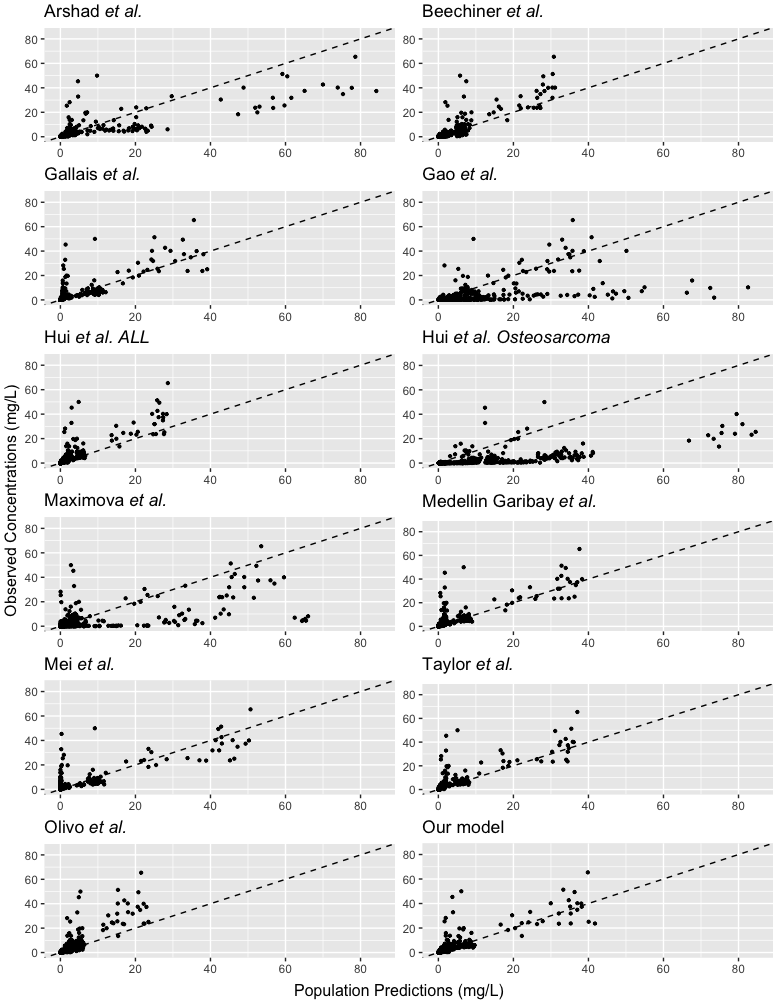
**

Abbreviation: ALL, acute lymphoblastic leukemia.

**Table S2: Predictive performance of models re-fit to test data**

| **Model** | **RMSE** | **MPE** | **MAPE** | **F20** | **F30** |
| --- | --- | --- | --- | --- | --- |
| Beechinor | 3.87 | 27.26 | 54.71 | 17.55 | 27.72 |
| Gallais | 5.33 | 26.18 | 51.37 | 22.28 | 33.01 |
| Hui ALL | 4.29 | 30.55 | 54.25 | 21.17 | 31.75 |
| Olivo | 5.49 | 15.20 | 45.75 | 21.17 | 32.45 |
| Taylor | 5.23 | 36.15 | 59.21 | 21.03 | 30.78 |
| Our final model | 5.91 | 10.61 | 46.88 | 24.51 | 35.52 |

Abbreviation: RMSE, root mean squared error; MPE, median percent prediction error; MAPE, median absolute percent prediction error; F20, proportion of percent prediction errors between -20% and 20%; F30, proportion of percent prediction errors between -30% and 30%; ALL, acute lymphoblastic leukemia.

**Table S3: Likelihood function values of re-fit models**

| **Model** | **OFV** | **AIC** | **BIC** |
| --- | --- | --- | --- |
| Beechinor | -832.72 | -816.72 | -790.14 |
| Gallais | -811.33 | -793.22 | -763.42 |
| Hui ALL | -865.62 | -841.62 | -801.74 |
| Olivo | -813.92 | -793.92 | -760.69 |
| Taylor | -572.70 | -548.70 | -508.82 |
| Our final model | -958.29 | -958.29 | -958.29 |

Abbreviation: OFV, objective function value; AIC, Aikaike information criterion; BIC, Bayesian information criterion; ALL, acute lymphoblastic leukemia.

**Figure S2: Boxplots of PE% and IPE%.** Full data range. The four panels represent: the first population-predicted concentration (top left); the Bayesian forecast of the second concentration using the first concentration as prior information (top right); the Bayesian forecast of the third concentration using the second concentration as prior information (bottom left); and the Bayesian forecast of the third concentration using the first and second concentrations as prior information (bottom right).


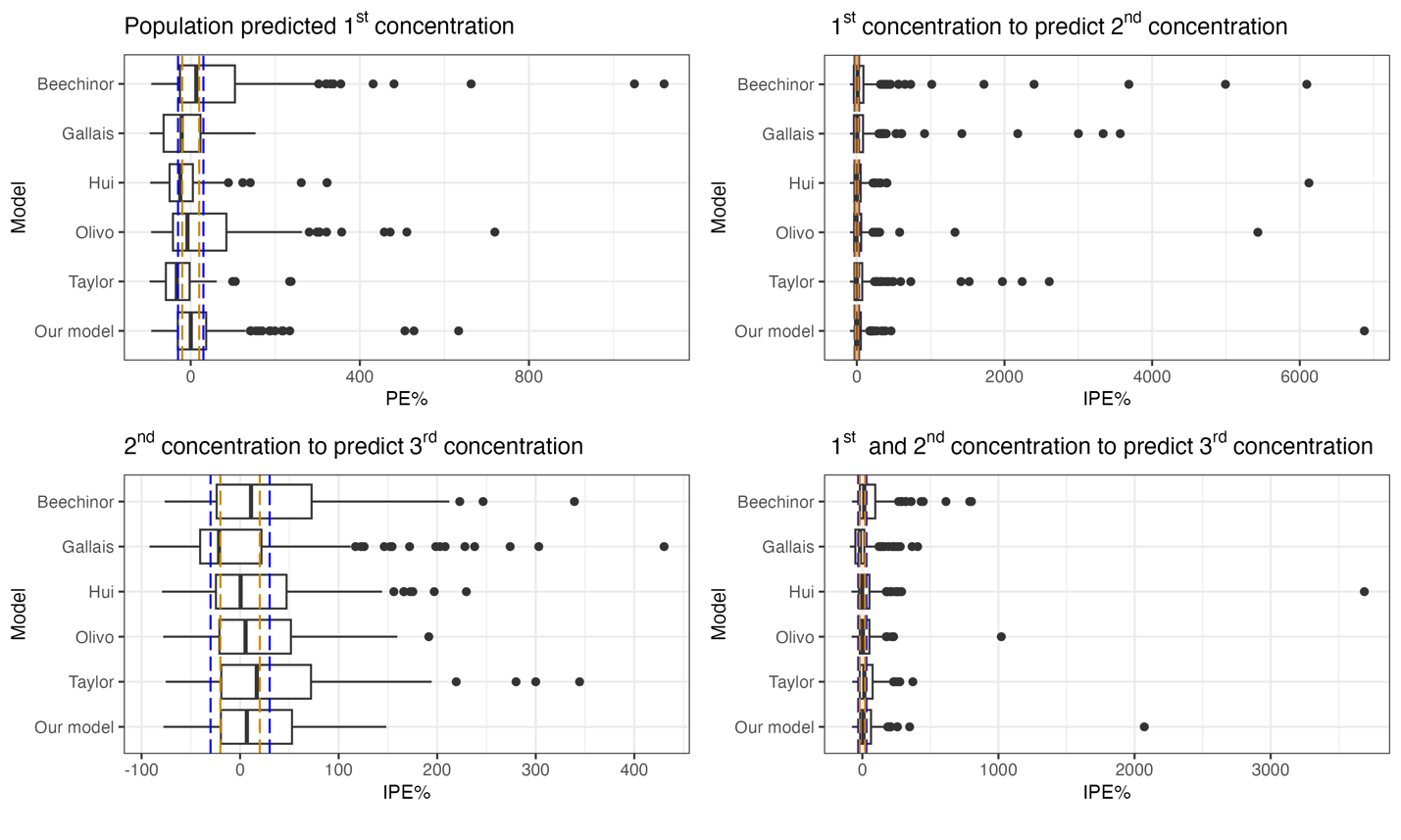


Note: All panels exclude outliers above 200%. Gold dashed lines at -20% and 20% visualize the IF20 and blue dashed lines at -30% and 30% visualize the IF30.

Abbreviation: PE%, percent prediction error; IPE%, individual percent prediction error; F20, proportion of percent prediction errors between -20% and 20%; F30, proportion of percent prediction errors between -30% and 30%.

**Table S4: Comparison of final model with and without sex**

| **Descriptor** | **With sex** | **Without sex** |
| --- | --- | --- |
| **Likelihood**  OFV  AIC  BIC | -2512.1  -2478.1  -2407.3 | -2486.4  -2454.4  -2387.8 |
| **PK parameters**  CL  V1  Q2  V2  Q3  V3  BSA exponent | 9.41  30.67  0.87  5.62  0.14  10.60  0.61 | 9.01  27.9  0.70  5.23  0.11  8.54  0.81 |
| **Overall fit**  RMSE  MPE  MAPE  F20  F30 | 5.91  10.61  46.88  24.51  35.52 | 6.24  14.72  47.18  25.63  36.21 |
| **Predicting 1^st^**  RMSE  MPE  MAPE  F20  F30 | 10.99  0.10  33.81  31.22  44.39 | 10.69  1.53  33.53  30.73  45.37 |
| **Predicting 2^nd^ with 1^st^**  RMSE  MPE  MAPE  F20  F30 | 1.02  23.76  48.54  24.35  35.23 | 1.06  27.38  51.10  25.91  34.72 |
| **Predicting 3^rd^ with 2^nd^**  RMSE  MPE  MAPE  F20  F30 | 0.08  9.58  37.50  26.16  38.95 | 0.08  2.83  37.29  24.42  39.53 |
| **Prediction 3^rd^ with 1^st^ and 2^nd^**  RMSE  MPE  MAPE  F20  F30 | 0.09  9.16  37.15  30.23  40.12 | 0.09  2.93  38.26  29.65  38.37 |

Abbreviation: OFV, objective function value; AIC, Aikaike information criterion; BIC, Bayesian information criterion; PK, pharmacokinetic; CL, clearance (L/hour); V1, volume of distribution in the main compartment (L); Q2, intercompartmental clearance between the main compartment and the peripheral compartment (L/hour); V2, volume of distribution in the peripheral compartment (L); Q3, intercompartmental clearance between the main compartment and the secondary peripheral compartment (L/hour); V3, and volume of distribution in the secondary peripheral compartment (L); BSA, body surface area; RMSE, root mean squared error; MPE, median percent prediction error; MAPE, median absolute percent prediction error; F20, proportion of percent prediction errors between -20% and 20%; F30, proportion of percent prediction errors between -30% and 30%.
